# Postoperative vaginal anatomy and dyspareunia after vNOTES hysterectomy without apical suspension: a comparative study

**DOI:** 10.3389/fsurg.2026.1934636

**Published:** 2026-10-19

**Authors:** Seyhmus Tunc, Kevser Arkan, Huseyin Kayaalp, Ali Deniz Erkmen, Gul Cavusoglu Colak, Sedat Akgöl

**Affiliations:** 1 Department of Obstetrics and Gynecology, Diyarbakir Gazi Yasargil Research and Training Hospital, Diyarbakir, Türkiye; 2 Division of Gynecologic Oncology, Department of Obstetrics and Gynecology, Diyarbakir Gazi Yasargil Research and Training Hospital, Diyarbakir, Türkiye; 3 Department of Obstetrics and Gynecology, Perinatology Clinic, Health Sciences University Ankara Bilkent City Hospital, Ankara, Türkiye

**Keywords:** apical support, dyspareunia, hysterectomy, sexual function, vaginal apex, vNOTES

## Abstract

**Objective:**

This study compared postoperative vaginal length, apical support, dyspareunia, and sexual function outcomes among vNOTES, laparoscopic, and abdominal hysterectomy in patients who did not undergo concomitant apical suspension.

**Methods:**

This retrospective comparative study included 140 women aged 40–55 years who underwent hysterectomy for benign indications between June 2018 and June 2024. Patients were allocated to three groups according to the surgical approach: vNOTES (*n* = 60), total laparoscopic hysterectomy (TLH; *n* = 40), and total abdominal hysterectomy (TAH; *n* = 40). None of the included patients had undergone concomitant apical suspension at the time of hysterectomy. The primary anatomical outcomes were vaginal length (VL) and apical support, assessed using the Pelvic Organ Prolapse Quantification (POP-Q) C-point, at 6 months postoperatively. Sexual function was assessed using the Female Sexual Function Index (FSFI), and dyspareunia severity was evaluated using a 10-cm visual analog scale (VAS).

**Results:**

Postoperative vaginal length was shortest in the vNOTES group (7.2 ± 0.6 cm), compared with the TLH (7.9 ± 0.7 cm) and TAH (8.4 ± 0.8 cm) groups (*p* < 0.001). Similarly, the C-point was located more caudally in the vNOTES group (−4.5 ± 1.0 cm) than in the TLH (−6.1 ± 0.9 cm) and TAH (−6.8 ± 1.0 cm) groups (*p* < 0.001). Significant differences in FSFI and dyspareunia scores were observed at 6 months, with lower FSFI and higher dyspareunia scores in the vNOTES group (*p* < 0.001).

**Conclusion:**

The surgical approach was associated with differences in postoperative vaginal anatomy and dyspareunia. Compared with TLH and TAH, vNOTES without apical suspension was associated with shorter vaginal length, a more caudal apical position, and higher dyspareunia scores. Because apical suspension was not directly evaluated, whether concomitant apical support would modify these outcomes remains unknown and warrants prospective investigation.

## 1 Introduction

Hysterectomy remains one of the most frequently performed procedures in gynecological surgery. In recent years, there has been a clear shift toward minimally invasive techniques, aiming to reduce perioperative morbidity and enhance recovery (1, 2). As surgical approaches have evolved from abdominal to laparoscopic and natural orifice transluminal endoscopic surgery (NOTES), the focus has extended beyond perioperative outcomes to include long-term anatomical and functional results that influence quality of life (3).

In sexually active women, preservation of vaginal anatomy, particularly vaginal length (VL) and apical support, is closely linked to postoperative sexual function (4). Vaginal shortening and downward displacement of the apex may contribute to dyspareunia and reduced sexual satisfaction (5, 6). Although sexual outcomes after hysterectomy are influenced by multiple factors, postoperative vaginal anatomy may vary according to the surgical approach (7).

The postoperative configuration of the vaginal apex is influenced by the level of colpotomy and the technique of cuff closure (8). In total abdominal hysterectomy (TAH), the wide surgical exposure allows a higher colpotomy and facilitates anatomical reconstruction, often preserving apical position (8). In total laparoscopic hysterectomy (TLH), uterine manipulators provide cephalad traction, enabling a controlled incision at the cervicovaginal junction under direct visualization (9).

Vaginal natural orifice transluminal endoscopic surgery (vNOTES) represents a further step in minimally invasive surgery, combining endoscopic visualization with a transvaginal approach (10). The increasing adoption of vNOTES is supported by its minimally invasive nature and potential clinical advantages, including avoidance of abdominal incisions and favorable postoperative recovery. These features make vNOTES an attractive alternative to conventional laparoscopic and abdominal approaches in appropriately selected patients. However, this technique introduces distinct mechanical conditions, including vaginal distension and sustained downward traction during uterine mobilization and extraction, which may be associated with differences in the final position of the vaginal apex (7, 10). Despite its increasing use, postoperative vaginal anatomy following vNOTES remains insufficiently characterized, particularly in the absence of routine apical suspension. This gap is clinically relevant, as preservation of apical support may be critical for maintaining vaginal length and reducing dyspareunia.

Current evidence remains inconsistent, often limited by heterogeneous study populations and variability in the use of apical support procedures such as McCall culdoplasty (10, 11). In addition, the clinical relevance of modest differences in vaginal length is still debated, with some authors emphasizing apical support rather than absolute length (7).

This study aimed to compare the three primary hysterectomy approaches in a well-defined cohort. By excluding patients with pre-existing prolapse and restricting the cohort to patients who had not undergone concomitant apical suspension, we sought to compare postoperative vaginal anatomy and sexual outcomes across vNOTES, TLH, and TAH. In doing so, we aimed to examine the association between surgical route and postoperative vaginal configuration and dyspareunia in the absence of additional apical reinforcement.

## 2 Materials and methods

### 2.1 Study population

This retrospective comparative study was conducted in the Department of Obstetrics and Gynecology at Gazi Yaşargil Training and Research Hospital. The study included patients who underwent hysterectomy for benign indications as part of routine clinical care between June 2018 and June 2024. Institutional Review Board approval was obtained (Approval No. 539, November 7, 2025), and all procedures were performed in accordance with institutional and national ethical standards. Written informed consent was obtained from all participants. Retrospective review of the medical records and extraction of data for research purposes were initiated after Institutional Review Board approval.

Medical records of patients who underwent hysterectomy for benign indications were reviewed. To ensure comparability between groups, the following inclusion criteria were applied: (1) premenopausal women aged 40-55 years, (2) sexually active status, (3) uterine weight < 500 g, and (4) pelvic organ prolapse stage 0–1 according to the pelvic organ prolapse quantification (POP-Q) system. Patients with a history of pelvic reconstructive surgery, deep infiltrating endometriosis, chronic pelvic pain syndrome, or suspected malignancy were excluded from the analysis.

A total of 140 eligible patients were stratified according to surgical approach: vNOTES (*n* = 60), TLH (*n* = 40), and TAH (*n* = 40). The surgical route was determined preoperatively on an individual basis according to patient characteristics, uterine size, previous pelvic or abdominal surgery, clinical assessment, surgeon experience, and patient preference. Surgical approach was not assigned for the purposes of this study, as the study was retrospective. To improve comparability across surgical groups, the same inclusion and exclusion criteria were applied to all three groups. Patients with factors likely to substantially increase surgical complexity, including uterine weight ≥500 g, POP-Q stage ≥2, deep infiltrating endometriosis, previous pelvic reconstructive surgery, or suspected malignancy, were excluded from the study. The groups were comparable in terms of age, parity, number of vaginal deliveries, and history of cesarean section. This approach was used to improve comparability among the surgical groups and reduce potential confounding.

All procedures were performed by senior surgeons experienced in each technique. Each participating surgeon had sufficient prior experience with the corresponding surgical technique before the study period. No missing data were identified for the primary anatomical outcomes or postoperative sexual function assessments included in the final analysis. Review of the operative records confirmed that none of the patients included in the study underwent a concomitant apical suspension procedure, such as McCall culdoplasty or uterosacral ligament suspension, at the time of hysterectomy (11). This approach allowed postoperative vaginal anatomy to be evaluated across surgical groups in the absence of concomitant apical suspension.

### 2.2 Surgical techniques

1. vNOTES group: Following placement of the vNOTES port, a circumferential colpotomy was performed. Caudal traction was applied during transvaginal uterine extraction using standard maneuvers. Vaginal cuff closure was completed transvaginally with a continuous absorbable 1-0 polyglactin 910 (Vicryl) suture.
2. TLH group: Cephalad counter-traction was achieved using a uterine manipulator. Colpotomy was performed laparoscopically at the cervicovaginal junction using electrosurgical energy. The vaginal cuff was closed laparoscopically with a barbed suture (V-Loc).
3. TAH group: Abdominal access was obtained via a Pfannenstiel incision. The vagina was transected at the level of the external cervical os, and the cuff was closed with interrupted absorbable 1-0 polyglactin 910 (Vicryl) sutures.

Colpotomy level and vaginal cuff closure were performed according to the standardized technique used for each surgical approach. In the TLH group, the uterine manipulator created a tenting effect, allowing colpotomy at the cervicovaginal junction. In the vNOTES group, colpotomy was performed at the most proximal level permitted by vaginal distension. Vaginal cuff closure was performed transvaginally with continuous absorbable sutures in the vNOTES group, laparoscopically with barbed sutures in the TLH group, and with interrupted absorbable sutures in the TAH group.

### 2.3 Anatomical and functional assessments

The primary anatomical outcomes were vaginal length (VL) and apical support at 6 months postoperatively. Vaginal length was measured as the distance from the hymenal ring to the vaginal apex using a calibrated rigid probe. Pelvic organ support was assessed by vaginal examination using the Pelvic Organ Prolapse Quantification (POP-Q) system. Measurements were recorded in centimeters relative to the hymen. The C-point represented the most distal edge of the vaginal cuff after hysterectomy and was used as a measure of apical position/support. More negative C-point values indicated a more cranial apical position, whereas less negative or positive values indicated a more caudal apical position. Postoperative evaluations were performed at 3 and 6 months; primary analyses were based on 6-month outcomes. All anatomical measurements were performed by the same investigator who was blinded to the surgical group. Secondary outcomes included sexual function, assessed using the Female Sexual Function Index (FSFI), and dyspareunia severity, evaluated using a 10-cm visual analog scale (VAS) (12, 13). For the purposes of this study, dyspareunia was defined as pain experienced during sexual intercourse and was assessed only in sexually active participants using a 10-cm VAS. The FSFI total score ranges from 2 to 36, with higher scores indicating better sexual function and lower scores indicating poorer sexual function. The VAS ranges from 0 (no pain) to 10 (worst imaginable pain), with higher scores indicating greater dyspareunia severity. The FSFI is a validated questionnaire assessing multiple domains of female sexual function. FSFI assessments were performed at 6 months postoperatively.

### 2.4 Statistical analysis

Statistical analyses were performed using SPSS version 25.0 (IBM Corp., Armonk, NY, USA). Normality of continuous variables was assessed using the Shapiro–Wilk test. Continuous variables are presented as mean ± standard deviation, and categorical variables as number and percentage. Differences in continuous variables among the three groups were analyzed using one-way analysis of variance (ANOVA), followed by Tukey's *post hoc* test for pairwise comparisons when appropriate. Categorical variables were compared using the Pearson chi-square test or Fisher's exact test, as appropriate. Effect sizes were estimated using eta-squared (*η*^2^) to quantify the magnitude of between-group differences in continuous outcomes. A two-sided *p* value < 0.05 was considered statistically significant. A *post hoc* power analysis was performed using G*Power version 3.1 (Heinrich Heine University, Düsseldorf, Germany). Assuming a one-way ANOVA, an alpha level of 0.05, and a total sample size of 140, the study achieved a *post hoc* power of >95% for the primary anatomical outcomes based on the observed large effect sizes (*η*^2^ = 0.37 for vaginal length and *η*^2^ = 0.49 for C-point position), indicating adequate statistical power to detect the observed between-group differences.

## 3 Results

The study population consisted of 140 patients who met the inclusion criteria. As shown in Table 1, there were no significant differences among the vNOTES (*n* = 60), TLH (*n* = 40), and TAH (*n* = 40) groups in terms of demographic and clinical characteristics. Mean age was similar across groups (48.2 ± 4.1, 47.9 ± 3.8, and 48.5 ± 4.5 years for vNOTES, TLH, and TAH, respectively; *p* = 0.82). The number of vaginal deliveries, cesarean sections, BMI, and uterine volume were also comparable between groups (all *p* > 0.05). The distribution of surgical indications did not differ significantly among the three groups (*p* = 0.98), and the corresponding frequencies and percentages are presented in Table 1.

**Table 1:** Demographic and clinical characteristics and surgical indications of the study groups.

Variable	Category	vNOTES (*n* = 60)	TLH (*n* = 40)	TAH (*n* = 40)	*p* value
Age (years)		48.2 ± 4.1	47.9 ± 3.8	48.5 ± 4.5	0.82
BMI (kg/m^2^)		27.4 ± 2.1	28.1 ± 2.5	27.8 ± 2.3	0.75
Parity		3.2 ± 1.1	3.3 ± 1.2	3.1 ± 1.0	0.68
Vaginal deliveries (*n*)		2.1 ± 0.7	2.2 ± 0.8	2.0 ± 0.6	0.75
Cesarean section (*n*)		1.1 ± 0.4	1.0 ± 0.3	1.2 ± 0.5	0.88
Uterine volume (cm^3^)		245 ± 50	255 ± 60	260 ± 55	0.68
Surgical indication (*n*/%)	Uterine leiomyoma	25 (41.7)	17 (42.5)	20 (50.0)	0.98
	Abnormal uterine bleeding	19 (31.7)	12 (30.0)	10 (25.0)	
	Adenomyosis	12 (20.0)	8 (20.0)	7 (17.5)	
	Other benign indications^a^	4 (6.7)	3 (7.5)	3 (7.5)	

Data are presented as mean ± standard deviation (SD) or number (*n*), as appropriate. BMI, body mass index; vNOTES, vaginal natural orifice transluminal endoscopic surgery; TLH, total laparoscopic hysterectomy; TAH, total abdominal hysterectomy. Continuous variables were compared using one-way ANOVA, and the distribution of surgical indications was compared using the Pearson chi-square test.

a Other benign indications included benign endometrial hyperplasia without atypia, persistent cervical dysplasia without suspected invasive malignancy, and benign adnexal masses accompanying uterine pathology, in strict accordance with the benign inclusion criteria.

### 3.1 Anatomical outcomes: vaginal length and apical support

At 6 months postoperatively, vaginal length (VL) differed significantly among the surgical groups (Table 2). Mean VL was 8.4 ± 0.8 cm in the TAH group, 7.9 ± 0.7 cm in the TLH group, and 7.2 ± 0.6 cm in the vNOTES group (*p* < 0.001*, η^2^* *=* 0.37). Vaginal length was significantly shorter in the vNOTES group compared with both TLH and TAH.

**Table 2:** Postoperative vaginal anatomical outcomes at 6 months.

Parameter	vNOTES (*n* = 60)	TLH (*n* = 40)	TAH (*n* = 40)	*p* value	Effect Size (*η*2)
Vaginal length (cm)	7.2 ± 0.6	7.9 ± 0.7	8.4 ± 0.8	< 0.001	0.37
C-point (POP-Q, cm)	−4.5 ± 1.0	−6.1 ± 0.9	−6.8 ± 1.0	< 0.001	0.49

Data are presented as mean ± standard deviation (SD). POP-Q, Pelvic Organ Prolapse Quantification system. More negative C-point values indicate a more cranial apical position. *P* values were calculated using one-way ANOVA followed by Tukey's *post hoc* test. $\eta^{2}$, eta-squared effect size.

Apical support, assessed by the POP-Q C-point, showed a similar pattern. The C-point was located more caudally in the vNOTES group (−4.5 ± 1.0 cm) compared with the TLH (−6.1 ± 0.9 cm) and TAH (−6.8 ± 1.0 cm) groups (*p* < 0.001, *η^2^* = 0.49).

### 3.2 Functional outcomes: sexual function and dyspareunia

At 6 months postoperatively, significant differences in FSFI scores were observed among the surgical groups (Table 3). At 6 months, the mean FSFI score was lower in the vNOTES group (26.0 ± 2.8) compared with TLH (28.5 ± 2.5) and TAH (29.4 ± 2.3) (*p* < 0.001, *η^2^* = 0.28).

**Table 3:** Postoperative sexual function and dyspareunia at 6 months.

Parameter	vNOTES (*n* = 60)	TLH (*n* = 40)	TAH (*n* = 40)	*p*-value	Effect Size (*η*2)
FSFI total score	26.0 ± 2.8	28.5 ± 2.5	29.4 ± 2.3	< 0.001	0.28
Dyspareunia (VAS, 0–10)	2.2 ± 0.9	1.7 ± 0.8	1.5 ± 0.7	< 0.001	0.15

Data are presented as mean ± standard deviation (SD). FSFI, Female Sexual Function Index; VAS, visual analog scale. Higher VAS scores indicate more severe dyspareunia. *P* values were obtained using one-way ANOVA with Tukey's *post hoc* test. $\eta^{2}$, eta-squared effect size.

Dyspareunia severity, assessed using VAS scores, was higher in the vNOTES group (2.2 ± 0.9) compared with TLH (1.7 ± 0.8) and TAH (1.5 ± 0.7) (*p* < 0.001, *η^2^* = 0.15).

## 4 Discussion

The present study identified significant associations between the surgical route of hysterectomy and postoperative vaginal anatomy and sexual outcomes. In this cohort, vNOTES was associated with a shorter vaginal length and a more caudal apical position compared with both laparoscopic and abdominal approaches. These anatomical differences were accompanied by higher dyspareunia scores and lower FSFI values at 6 months. Together, these findings demonstrate concurrent differences in postoperative anatomical and functional outcomes among the surgical groups, although their causal and clinical relationships cannot be established from the present retrospective data (7, 14, 15).

Previous studies examining the effect of hysterectomy on vaginal length and sexual function have reported inconsistent findings, largely due to differences in patient selection and surgical techniques (3, 6). In many reports, the use of adjunctive apical support procedures may obscure the association between the surgical route and postoperative outcomes (11). In the present study, none of the included patients had undergone concomitant apical suspension, allowing comparison of anatomical outcomes across surgical approaches without the potential influence of an additional apical support procedure. However, because apical suspension was not performed or directly evaluated in any group, this study cannot determine whether concomitant apical suspension would have prevented or modified the observed postoperative outcomes. Under these conditions, a consistent pattern was observed, with TAH associated with the greatest vaginal length, followed by TLH, while vNOTES showed the shortest measurements.

We suggest that differences in traction patterns between surgical approaches may account for the observed anatomical variations. In TLH, cephalad counter-traction provided by the uterine manipulator facilitates colpotomy at a higher level, whereas vNOTES relies on downward traction, which may result in a more distal incision and a more caudal apical position. In addition, vaginal distension during vNOTES may influence tissue tension at the time of cuff closure. These factors may contribute to reduced vaginal length and higher dyspareunia scores observed in the vNOTES group although a direct causal relationship cannot be established. Our findings also highlight the distinction between static anatomical measurements and functional anatomy. While differences in vaginal length may appear modest at rest, their impact may become more pronounced during functional states such as intercourse, where tissue elasticity and apical stability are required. Furthermore, the calculated effect sizes for both VL (*η^2^* = 0.37) and C-point position (*η^2^* = 0.49) demonstrated a large effect according to Cohen's criteria (where *η^2^* ≥ 0.14 indicates a large effect). These large effect sizes indicate that the observed anatomical differences were not only statistically significant but also of substantial magnitude.

The functional findings support this pattern. At the 6-month evaluation, the vNOTES cohort demonstrated lower FSFI scores and higher dyspareunia scores (VAS 2.2 ± 0.9) than the TLH and TAH groups. Although these between-group differences were statistically significant, their clinical relevance should be interpreted cautiously. Because validated minimal clinically important difference thresholds for the FSFI and dyspareunia VAS in this specific postoperative population were not prespecified, and preoperative scores were unavailable, the present data do not allow us to determine whether the observed differences exceed a clinically meaningful threshold. Nevertheless, even modest anatomical differences in vaginal length may have functional relevance during intercourse, where vaginal distensibility and apical support are required (16, 17). This is in line with previous studies emphasizing the importance of apical support in maintaining sexual function (4, 17).

The clinical implication of these findings is not that vNOTES is inferior, but that its technical aspects require careful consideration. The advantages of vNOTES, including faster recovery and the absence of abdominal scars, remain clear (14, 18). However, our results demonstrate differences in postoperative vaginal anatomy among the surgical groups in the absence of concomitant apical support. Because apical suspension was not evaluated in this study, whether adjunctive apical support during vNOTES could help preserve postoperative vaginal anatomy remains unknown and requires evaluation in prospective comparative studies (19, 20).

A key strength of this study is the use of a well-defined cohort. By restricting the age range and excluding patients with pre-existing prolapse, we aimed to reduce confounding factors and allow a clearer assessment of the association between surgical approach and postoperative outcomes. The main limitations include the retrospective single-center design and the non-randomized selection of the surgical route, which may have introduced residual selection bias despite the use of common inclusion and exclusion criteria across groups. Accordingly, the observed between-group differences should be interpreted as associations rather than causal effects of the surgical approach. Differences in vaginal cuff closure technique and suture material among the surgical groups represent a potential procedural confounder. Specifically, cuff closure differed with respect to the route of closure, suture technique, and suture material across the vNOTES, TLH, and TAH groups. These technical differences may be associated with postoperative vaginal length and apical configuration; therefore, the observed anatomical differences cannot be attributed solely to the surgical route. Because cuff-closure techniques were not standardized across groups, their independent contribution to the observed outcomes could not be determined in this retrospective study. In addition, preoperative sexual function and dyspareunia data were unavailable; therefore, postoperative between-group differences in FSFI and dyspareunia could not be interpreted as changes from individual preoperative baseline values. The follow-up period was limited to 6 months. Although functional outcomes are generally stable by this time, longer follow-up is needed to assess potential long-term implications, including pelvic floor support.

### 4.1 Refining the vNOTES technique: practical considerations

Our findings raise the possibility that technical factors during vNOTES may be associated with postoperative vaginal anatomy. The potential relevance of colpotomy level and traction patterns warrants further investigation. Similarly, the potential role of concomitant apical suspension in postoperative apical support remains uncertain and should be evaluated in prospective comparative studies.

## 5 Conclusion

In this retrospective observational study, vNOTES hysterectomy without concomitant apical suspension was associated with shorter postoperative vaginal length, a more caudal apical position, and less favorable postoperative sexual-function measures compared with TLH and TAH. However, these findings should be interpreted as associations rather than causal effects of the surgical approach. Because apical suspension was not directly evaluated, this study cannot determine whether concomitant apical suspension would prevent or modify the observed outcomes. In addition, differences in vaginal cuff-closure techniques among the surgical groups and the absence of preoperative sexual-function and dyspareunia data should be considered when interpreting these findings. Prospective studies with standardized surgical techniques and preoperative and postoperative assessments are needed to confirm these associations and further evaluate their clinical implications.

## Data Availability

The data analyzed in this study is subject to the following licenses/restrictions: the datasets generated and/or analyzed during the current study are not publicly available due to patient privacy and ethical restrictions but may be available from the corresponding author upon reasonable request and subject to institutional approval. Requests to access these datasets should be directed to ST, drseyhmustunc@hotmail.com.
